# To predict the spread through air spaces in lung adenocarcinoma using radiomic features from different regions of part-solid nodules: a multicenter study

**DOI:** 10.3389/fonc.2025.1700843

**Published:** 2025-10-31

**Authors:** Shiyu Cui, Hongzheng Song, Fanxia Lin, Xiaomeng Han, Bo Wang, Liang Zhang, Feng Hou, Enhao Kang, Jizheng Lin, Henan Lou

**Affiliations:** ^1^ Department of Radiology, The Affiliated Hospital of Qingdao University, Qingdao, China; ^2^ Department of Radiology, Qingdao Municipal Hospital, Qingdao, China; ^3^ Department of Radiology, People’s Hospital of Rizhao, Rizhao, China; ^4^ Department of Nuclear Medicine, The Affiliated Hospital of Qingdao University, Qingdao, China; ^5^ Department of Pathology, The Affiliated Hospital of Qingdao University, Qingdao, China

**Keywords:** lung adenocarcinoma, spread through air spaces, part-solid nodules, tomography, X-ray computed, radiomics

## Abstract

**Background:**

This study aims to explore the value of radiomic features from different regions of part-solid nodules (PSNs) for predicting spread through air spaces (STAS) in lung adenocarcinoma.

**Methods:**

This retrospective analysis included 333 patients with PSNs lung adenocarcinoma pathologically confirmed in three hospitals. Data from one institution were utilized for training set (n=223), while the remaining two served as the external test set (n=110). The computed tomography radiomic features were extracted from different areas of the nodule (ground-glass, solid, gross, and perinodular). Three machine learning classifiers (support vector machine, light gradient boosting machine [LightGBM], logistic regression) were used to build predictive models. Model performance was assessed using accuracy and area under the curve (AUC). The DeLong test was used to determine differences in AUC values between models. The clinical benefits of models were assessed using decision curve analysis (DCA).

**Results:**

In the external test set, the radiomics model developed using combined features from ground-glass, solid, and perinodular regions with LightGBM classifier achieved an AUC of 0.840 (95% confidence interval [CI]: 0.758–0.921), which was better than the clinical model (AUC = 0.622, 95% CI: 0.494–0.750, P < 0.001) and other radiomics models. DCA indicated that this model has achieved a higher net benefit.

**Conclusion:**

The radiomics model developed using radiomic features of distinct solid and ground-glass components of PSNs and the perinodular region can contribute to identifying the STAS status in lung adenocarcinoma.

## Introduction

Lung cancer ranks among the most prevalent cancers globally, having the highest incidence rate (1). In most countries, adenocarcinoma emerges as the predominant pathological type, accounting for nearly 50% of all lung cancers (2). The detection rates for pulmonary nodules and early lung cancer have increased with the extensive application of low-dose chest computed tomography (CT) (3, 4). Pulmonary nodules can be classified according to CT findings into pure ground glass nodules, solid nodules, and part-solid nodules (PSNs).

Spread through air spaces (STAS) was recognized as an invasion mode of lung adenocarcinoma by the World Health Organization (WHO) in 2015. STAS refers to the spreading of micropapillary clusters, solid nests, or single cells beyond the edge of the tumor into the air space in the surrounding lung parenchyma (5). Lung adenocarcinoma with STAS shows a poor prognosis, with reduced overall survival and disease-free survival rates (6, 7). In recent years, sublobar resection has been widely used as a minimally invasive surgical method to treat early lung cancer (8, 9). However, lung cancer patients who exhibit STAS after sublobar resection have an increased risk of recurrence (10, 11). STAS serves as an important prognostic factor after sublobar resection for early lung adenocarcinoma, and such tumors may not be suitable for sublobar resection (12, 13). Unfortunately, STAS can only be determined by surgical methods at present, and there are still uncertainties regarding the accuracy of intraoperative frozen sections (14, 15). Therefore, it is very important to determine the status of STAS before operation because it helps clinicians choose the most appropriate surgical approach.

Previous research (16–18) showed that STAS mostly occurs in solid or part-solid nodules, whereas it is rarely observed in pure ground glass nodules. Compared with pure ground glass nodules, PSNs exhibit a high positive of STAS, along with a high invasiveness and a less favorable prognosis. Meanwhile, lung adenocarcinoma presenting as PSN is a special clinical subtype that can show different clinicopathological features from solid tumors (19). Consequently, special attention should be paid to PSNs.

Radiomics analysis using quantitative features extracted from medical images allows precise and detailed evaluation of lesions, including the presence of tumor heterogeneity (20). Several studies (21–24) have used radiomics method to assess STAS status in lung adenocarcinoma, and these have achieved good diagnostic performance. However, their study failed to offer a detailed analysis of lung adenocarcinoma with PSNs. Additionally, because of unclear internal mechanisms that limit transparency and credibility, the application of such models in clinical practice may be restricted (25). Shapley Additive exPlanations (SHAP) is a unified structure based on additive feature mapping techniques that consider the predictions of complex models (26). It can explain the importance of features and assist in comprehending the function of each feature in making predictions for both the entire dataset and specific samples (27). By combining radiomics and SHAP, it is possible to build a model that explains the prediction in an understandable way (28, 29).

This study aimed to construct and evaluate radiomics signatures derived from various areas of the nodule (ground-glass, solid, gross, and perinodular) for predicting STAS status in lung adenocarcinoma with PSNs. Moreover, we used the SHAP method to illustrate the decision-making process of the models and gain insights into the connections between radiomic features and STAS.

## Methods

### Patients

The Ethics Committee of our institution authorized this retrospective study (No. QYFY WZLL 29455) and waived the need for informed consent. Patients with lung adenocarcinoma who had undergone surgical resection in three hospitals between December 2019 and April 2024 were retrospectively collected. The inclusion criteria included. (1) pathology-confirmed invasive lung adenocarcinoma; (2) thin-slice CT examination (slice thickness ≤ 1.25 mm) performed within 1 month before operation; (3) tumor that was PSN with a maximum diameter ≤ 3 cm; and (4) clinicopathological data were complete. The exclusion criteria included: (1) patients with multiple lesions; (2) patients who received preoperative anti-tumor treatment (immunotherapy, chemotherapy, or radiotherapy); (3)patients who had previously been diagnosed with other malignant tumors; and (4) patients with low-quality image.

**Figure 1:**
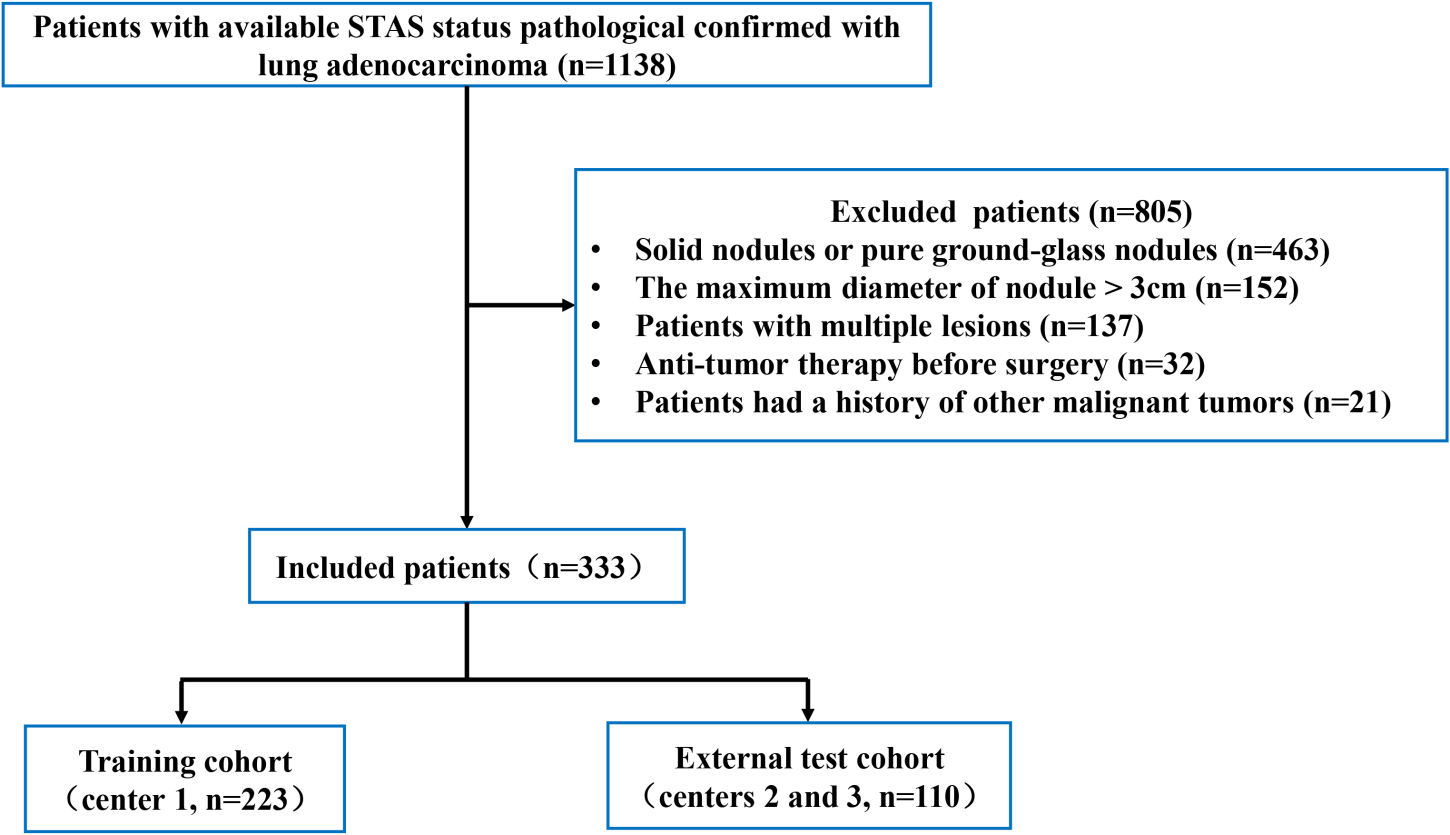
The process used to select patients is shown in the flowchart. STAS, spread through air spaces.

In total, 333 patients were collected (**Figure 1**). Patients were separated into a training group (n=223, center 1) and an external test group (n=110, centers 2 and 3).

### Histopathologic evaluation

Two pathologists, unaware of the clinical outcomes for the patients, independently evaluated the tumor slides. Where any discrepancies, a consensus was achieved through discussion. According to the WHO classification, STAS refers to the existence of tumor cells in the lung air spaces beyond the margin of the main tumor. It has three main forms: (1) single cells, multiple separate and non-continuous single cells occupy the air spaces; (2) solid nests, where the solid component of the tumor fills the air spaces; and (3) micropapillary clusters, micro-nipple structures without central fibrovascular cores fill the air spaces (10, 30).

### Image acquisition

**Supplementary Table S1** outlines the parameters used for CT scanning. Unenhanced CT was acquired using a slice thickness of ≤ 1.25 mm.

### Clinical data collection and CT image evaluation

The clinical characteristics and CT findings of patients were analyzed, including gender, age, smoking history, consolidation/tumor ratio (CTR), maximum solid component diameter (Dsolid), maximum tumor diameter (Dmax), clinical T stage, carcinoembryonic antigen (CEA) level, nodule location, boundary, spiculation, lobulation, vascular convergence, pleural indentation, air bronchogram, and vacuole. Two experienced radiologists evaluated the CT images of the lesions. They did not know the pathological results of the lesions before evaluation and reached a consensus through discussion when there were differences in their evaluation results.

### Image segmentation and extraction of radiomic features

**Figure 2:**
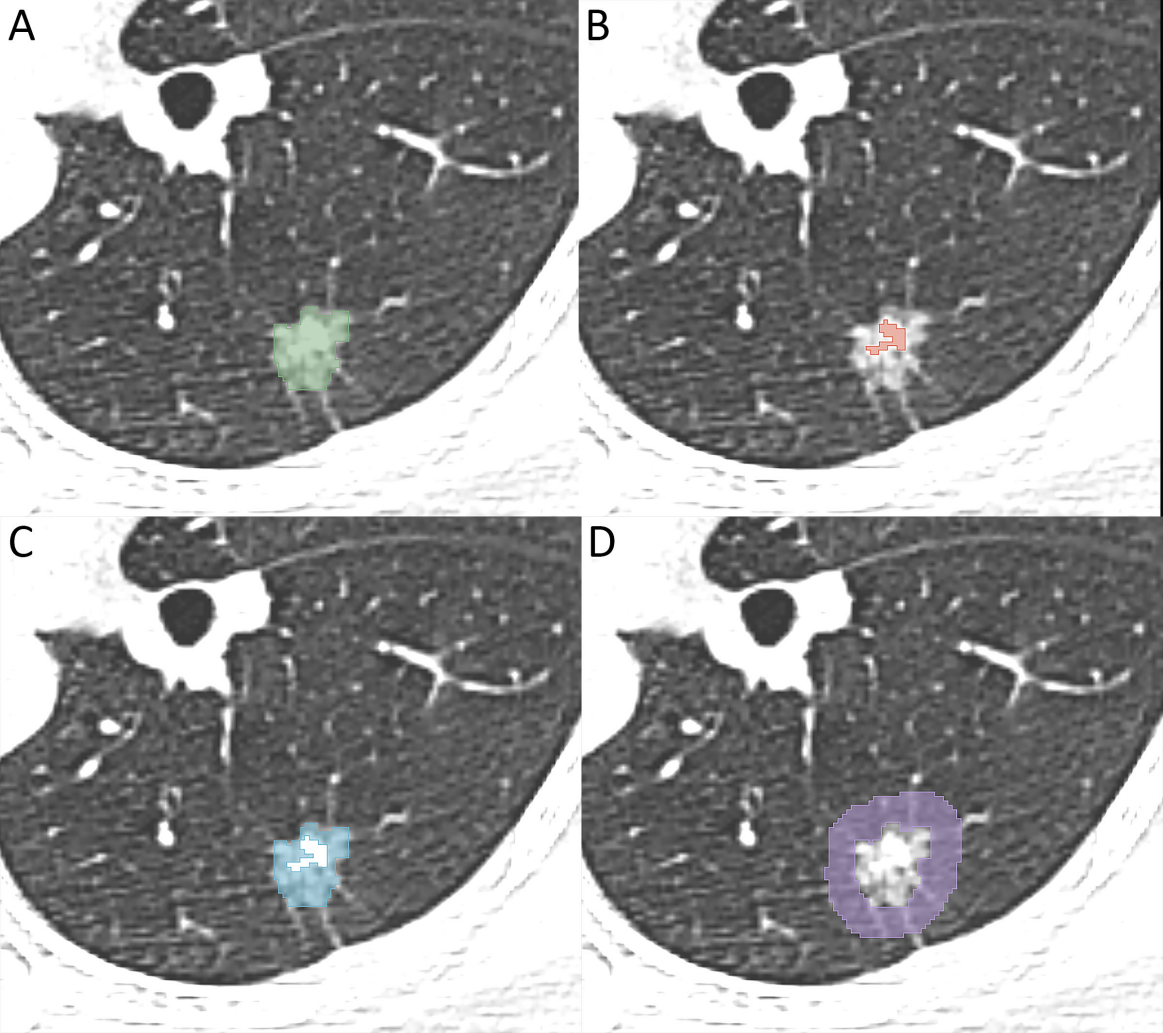
Image illustrates segmentation of different region of interest. **(A)** the gross nodule region, **(B)** the solid region, **(C)** the ground-glass opacity region, **(D)** the perinodular region.

An experienced radiologist manually delineated the regions of interest (ROIs) using 3D-slicer software (version 5.2.1, https://www.slicer.org). The gross nodule region (GNR), solid region (SR), ground-glass opacity region (GGR), and perinodular region (PR) were delineated as shown in **Figure 2**, and three-dimensional ROIs of the different nodule regions were generated. The segmentation steps were as follows: (1) the GNR was delineated around the edge of the nodule using the lung window (window level, -700 HU; window width, 1200 HU), excluding large bronchi and vessels as much as possible; (2) the SR was identified within the GNR by applying a thresholding method (> −50HU); (3) the GGR was obtained by subtracting the SR from the GNR; and (4) the PR was defined as extending 5 mm from the edge of the nodule to the periphery, excluding nearby soft tissues such as the mediastinum or chest wall (31).

Thirty lesions were randomly selected and delineated two weeks later by the same radiologist and another radiologist to allow intra- and inter-observer correlation coefficients (ICCs) to be computed. The radiologists were blinded to clinical and pathological data during the segmentation process.

Pyradiomics software (version 3.1.0) was used to extract radiomic features from the ROIs. To mitigate the impact of different CT spatial resolutions, all images were resampled to a voxel size of 1 × 1 × 1 mm. Finally, each ROI yielded a total of 1316 features, including a suite of texture features, 14 shape-based features, and 252 first-order features. The texture features consisted of 70 neighboring grey tone difference matrix (NGTDM) features, 196 grey-level dependence matrix (GLDM) features, 224 grey-level size zone matrix (GLSZM) features, 336 gray-level co-occurrence matrix (GLCM) features, and 224 grey-level run-length matrix (GLRLM) features.

### Selection of radiomic features and model construction

Features with ICCs > 0.75 were chosen for further analyses. All features were processed with Z-score normalization, and the combat compensation technique was employed to adjust those radiomic features that were influenced by batch effects resulting from different devices (32). The Spearman rank test was used to evaluate the correlation between features, and when the linear correlation coefficient was > 0.80, features were considered redundant and removed. Least absolute shrinkage and selection operator (LASSO) regression was then used to identify the features with the most predictive value. A total of 3 machine learning classifiers were used to construct models for the radiomic features from different regions of the nodule (GNR, SR, GGR, PR). These three classifiers were: support vector machine (SVM), light gradient boosting machine (LightGBM), logistic regression (LR). The classifiers were trained on the training set using a 10-fold cross-validation method.

### Clinical model construction

Univariate logistic regression analysis was used to identify variables associated with STAS status. Variables with P < 0.05 were further analyzed using multivariate logistic regression analysis. Variables yielding P < 0.05 in the multivariate analysis were deemed independent predictors of STAS. Using these significant variables, a clinical model was developed.

### Interpretability of the model using SHAP

SHAP technology was used to clarify and analyze the radiomic features applied to the radiomics models. This approach allows the significance of each feature in a machine learning model to be represented and provides a comprehensive explanation of how each feature affects the output result, either raising or lowering it.

**Figure 3:**
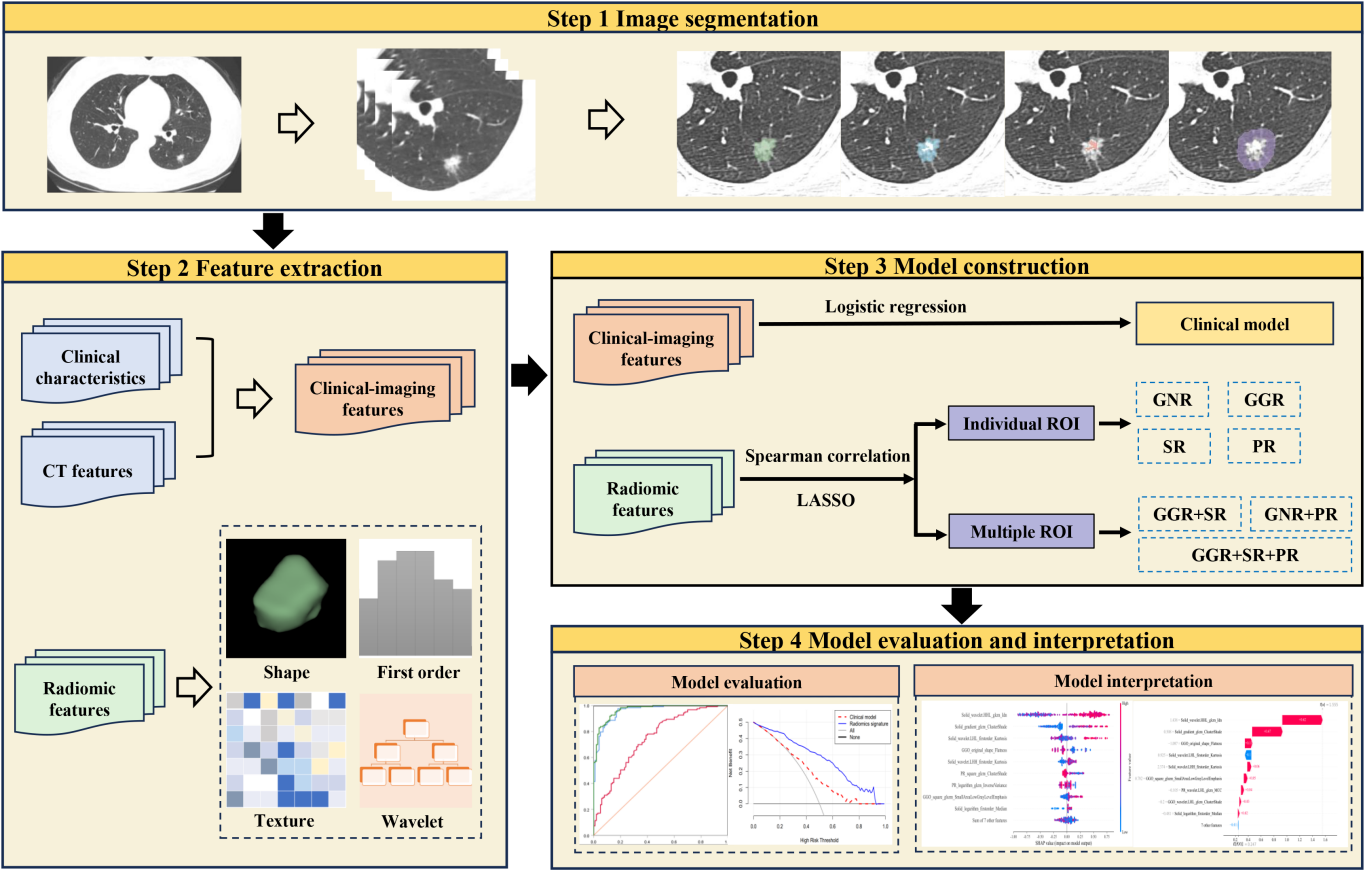
Flowchart of the study.

The SHAP summary plot can effectively visualize and interpret the significance of features in relation to the predictions of a model, with features being listed top-down on the basis of their importance. Compared with the bottom features, the top features exhibit greater contributions to the model and possess higher predictive power. The SHAP values were computed for the chosen radiomic features contained in the radiomics model showing the best performance. The SHAP value of a specific feature from an individual patient is represented by a dot, and these dots are stacked vertically and arranged horizontally to illustrate the density of identical SHAP values. Subsequently, each point is assigned a color based on the feature’s value. The SHAP force plot enables the evaluation of a single patient to be interpreted. The percentage contribution of a specific feature to the SHAP value is represented by the length of the arrow. Positive (red) or negative (blue) contributions are indicated by the color of the arrow. **Figure 3** illustrates the workflow of the study.

### Statistical analysis

Data were analyzed using SPSS software (version 26.0, IBM) and R software (version 4.3.1, www.r-project.org). Python (3.9.7, www.python.org) was used to build the machine learning models. The Kolmogorov-Smirnov test was used to test continuous data for normality. Comparative data analysis was conducted using the Mann-Whitney U test for non-normally distributed continuous data, independent samples *t*-tests was used for normally distributed continuous data, and Fisher’s exact test or the chi-square test were used for categorical variables.

The ability of the models to predict STAS status was assessed using the receiver operating characteristics (ROC) curve and the area under the curve (AUC), with a 95% confidence interval (CI) provided. The AUC values were compared between the best-performing model and the other models using the DeLong test. The clinical utility of the models was assessed using decision curve analysis (DCA). A value of P < 0.05 was considered statistically significant.

## Results

### Clinical and CT characteristics

**Table 1** provides details on the clinical and CT features of the patients. Among 333 patients with lung adenocarcinoma, 152 cases were STAS-positive, and 181 were STAS-negative. There were 210 women and 123 men, ranging in age from 29 to 83 years.

**Table 1:** Clinical and CT characteristics of patients.

Variable	STAS negative	STAS positive	P value
	(n=181)	(n=152)	
Age (y)	59.00 (53.00, 66.00)	59.00 (53.25, 67.00)	0.321
Sex			0.182
Female	120 (66.3%)	90 (59.2%)	
Male	61 (33.7%)	62 (40.8%)	
Smoking history			0.021
Never	153 (84.5%)	113 (74.3%)	
Former or current	28 (15.5%)	39 (25.7%)	
Nodule location			0.342
RUL	58 (32.0%)	43 (28.3%)	
RML	18 (9.9%)	9 (5.9%)	
RLL	26 (14.4%)	33 (21.7%)	
LUL	42 (23.2%)	36 (23.7%)	
LLL	37 (20.4%)	31 (20.4%)	
CEA			0.001
Normal	166 (91.7%)	120 (78.9%)	
Abnormal	15 (8.3%)	32 (21.1%)	
T stage			<0.001
T1a	84 (46.4%)	31 (20.4%)	
T1b	89 (49.2%)	93 (61.2%)	
T1c	8 (4.4%)	28 (18.4%)	
Dmax (mm)	16.00 (12.00, 20.00)	19.50 (15.00, 25.00)	<0.001
Dsolid (mm)	8.00 (5.00, 11.00)	13.00 (10.00, 18.00)	<0.001
CTR	0.55 (0.39, 0.72)	0.73 (0.56, 0.87)	<0.001
Boundary			0.256
Clear	79 (43.6%)	57 (37.5%)	
Unclear	102 (56.4%)	95 (62.5%)	
Lobulation			<0.001
No	70 (38.7%)	21 (13.8%)	
Yes	111 (61.3%)	131 (86.2%)	
Spiculation			<0.001
No	107 (59.1%)	40 (26.3%)	
Yes	74 (40.9%)	112 (73.7%)	
Pleural indentation			0.098
No	59 (32.6%)	37 (24.3%)	
Yes	122 (67.4%)	115 (75.7%)	
Air bronchogram			0.016
No	78 (43.1%)	46 (30.3%)	
Yes	103 (56.9%)	106 (69.7%)	
Vacuole			0.294
No	95 (52.5%)	71 (46.7%)	
Yes	86 (47.5%)	81 (53.3%)	
Vascular convergence			<0.001
No	83 (45.9%)	36 (23.7%)	
Yes	98 (54.1%)	116 (76.3%)	

RUL, right upper lobe; RML, right middle lobe; RLL, right lower lobe; LUL, left upper lobe; LLL, left lower lobe; CEA, carcinoembryonic antigen; CTR, consolidation/tumor ratio; STAS, spread through air spaces; Dmax, maximum tumor diameter; Dsolid, maximum solid component diameter.

Statistically significant differences were observed in smoking history, CEA, T stage, CTR, Dsolid, Dmax, lobulation, spiculation, air bronchogram, and vascular convergence between the STAS-positive and STAS-negative groups.

### Construction of the clinical model

When applied to the training set, the univariate logistic regression analysis demonstrated that risk factors predicting STAS in lung adenocarcinoma included CEA, T stage, Dsolid, Dmax, CTR, boundary, lobulation, spiculation, and vascular convergence (**Table 2**). Multivariate logistic regression analysis demonstrated that CTR was an independent predictor of STAS, and then the clinical model was built. Finally, the clinical model achieved an AUC value of 0.681 (95% CI: 0.611–0.752) for the training set and 0.622 (95% CI: 0.494–0.750) for the external test set (**Table 3**).

**Table 2:** Analysis by logistic regression of clinical and CT characteristics.

Variable	Univariate logistic analysis		Multivariate logistic analysis	
	OR (95% CI)	P value	OR (95% CI)	P value
Age	1.013 (0.987-1.041)	0.330		
Sex	1.256 (0.722-2.183)	0.420		
Smoking history	1.281 (0.659-2.492)	0.465		
Nodule location	0.998 (0.838-1.838)	0.983		
CEA	2.471 (1.194-5.115)	0.015	2.078 (0.907-4.765)	0.084
T stage	3.689 (2.288-5.946)	<0.001	2.075 (0.685-6.290)	0.197
Dmax	1.080 (1.031-1.131)	0.001	1.151 (0.961-1.380)	0.127
Dsolid	1.151 (1.090-1.215)	<0.001	0.803 (0.616-1.047)	0.105
CTR	29.502 (7.111-122.395)	<0.001	316.283 (1.296-77210.826)	0.040
Boundary	1.955 (1.142-3.346)	0.015	1.197 (0.637-2.248)	0.577
Lobulation	4.143 (1.673-10.258)	0.002	1.100 (0.375-3.223)	0.863
Spiculation	3.393 (1.908-6.033)	<0.001	1.819 (0.914-3.619)	0.088
Pleural indentation	1.691 (0.901-3.172)	0.102		
Air bronchogram	1.258 (0.719-2.202)	0.421		
Vacuole	1.093 (0.645-1.853)	0.742		
Vascular convergence	3.114 (1.758-5.517)	<0.001	1.736 (0.837-3.599)	0.138

OR, odds ratio; CI, confidence interval; CEA, carcinoembryonic antigen; CTR, consolidation/tumor ratio; Dmax, maximum tumor diameter; Dsolid, maximum solid component diameter.

**Table 3:** Diagnostic value of clinical model and each best machine learning model based on different nodule regions.

Model	Training cohort			External test cohort		
	AUC (95% CI)	Accuracy	P value	AUC (95% CI)	Accuracy	P value
Clinical model	0.681 (0.611-0.752)	0.664	<0.001	0.622 (0.494-0.750)	0.718	<0.001
GNR	0.765 (0.703-0.827)	0.713	<0.001	0.674 (0.561-0.787)	0.691	<0.001
SR	0.926 (0.894-0.958)	0.834	<0.001	0.831 (0.741-0.920)	0.755	0.752
GGR	0.738 (0.671-0.805)	0.704	<0.001	0.659 (0.540-0.779)	0.691	<0.001
PR	0.917 (0.880-0.953)	0.865	0.250	0.648 (0.523-0.773)	0.682	0.003
GGR+SR	0.936 (0.907-0.966)	0.857	0.007	0.832 (0.743-0.920)	0.836	0.646
GNR+PR	0.801 (0.744-0.859)	0.749	<0.001	0.714 (0.603-0.825)	0.600	<0.001
GGR+SR+PR	0.959 (0.936-0.982)	0.901	Reference	0.840 (0.758-0.921)	0.836	Reference

AUC, area under the receiver operating characteristic curve; CI, confidence interval; GNR, gross nodule region; SR, solid region; GGR, ground-glass opacity region; PR, perinodular region. The logistic regression classifier produced the best predictive performance in the GNR and GNR+PR models. The support vector machine classifier produced the best predictive performance in the GGR model. The light gradient boosting machine classifier produced the best predictive performance in the SR, PR, GGR+SR and GGR+SR+PR models.

### Construction of radiomics signatures and evaluation of their performance

**Figure 4:**
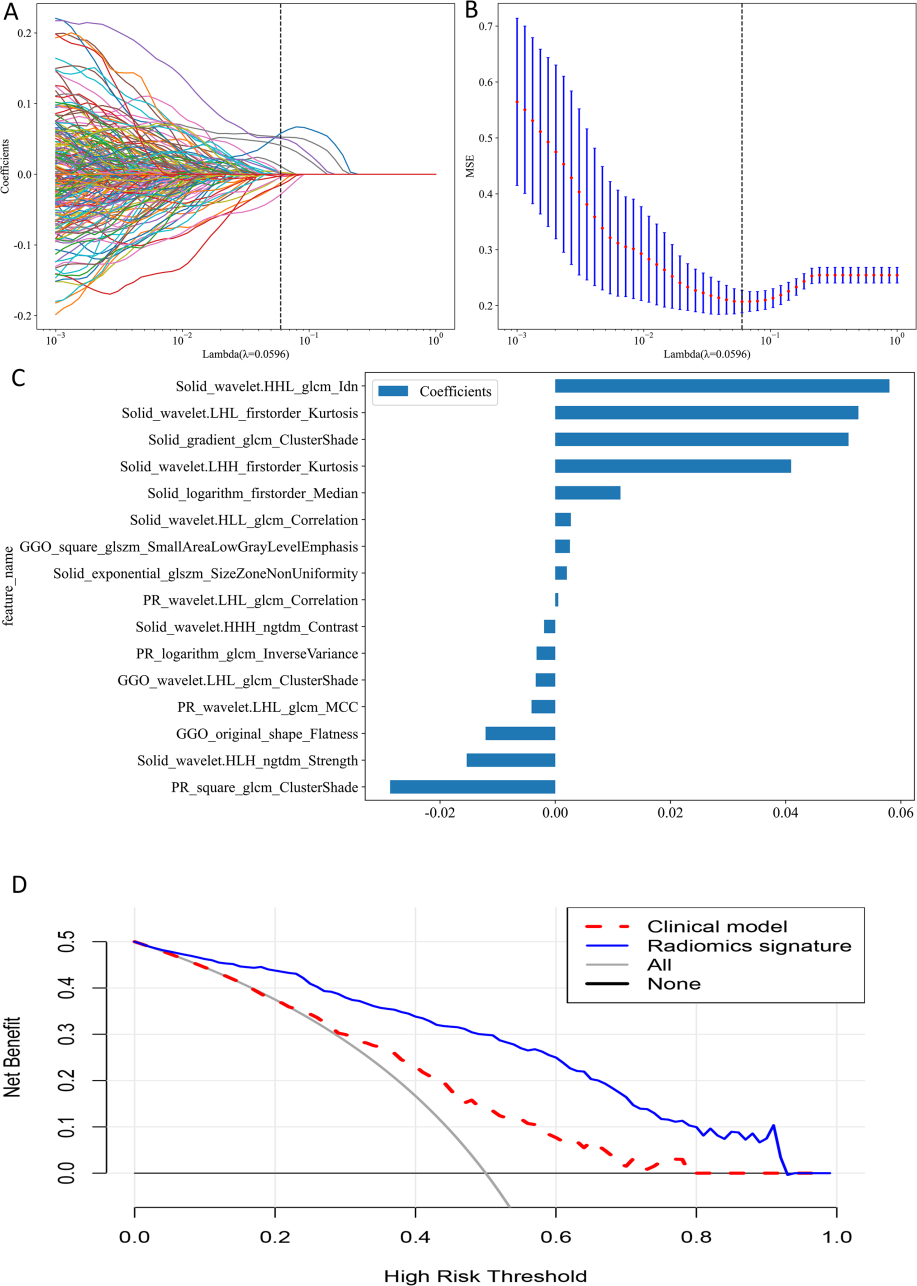
**(A)** The plot of coefficient profile. **(B)** The plot of cross-validation. **(C)** Feature weight histogram in best-performing model. **(D)** Decision curve analysis.

For individual ROIs (GNR, GGR, SR and PR), features with ICCs > 0.75 were retained, and further feature selection was carried out in the training group using Spearman correlation coefficients and the LASSO algorithm (**Figures 4A, B**). We identified 3, 4, 8, and 15 radiomic features with the highest predictive value for GNR, GGR, SR, and PR, respectively. We used the aforementioned 3 machine learning classifiers to establish radiomics signatures for the four ROIs. For both the training and external test groups, the LightGBM classifier model based on SR features (SR model) produced the highest predictive performance, with accuracy values of 0.834 and 0.755, respectively, and AUC values of 0.926 (95% CI: 0.894–0.958) and 0.831 (95% CI: 0.741–0.920) (**Table 3**). **Supplementary Table S2** provides details on the performance of three machine learning classifiers using radiomic features from individual ROIs.

For analysis of multiple ROIs (GGR+SR, GNR+PR and GGR+SR+PR), we combined the features of the selected ROIs before performing correlation analysis and applying the aforementioned screening methods, choosing 8, 7, and 16 radiomic features to construct the respective multiple ROI-based radiomics signatures. The LightGBM classifier model utilizing combined features from GGR, SR, and PR (GGR+SR+PR model) produced the greatest predictive performance (training group: AUC = 0.959, 95% CI: 0.936–0.982, Accuracy = 0.901; external test group: AUC = 0.840, 95% CI: 0.758–0.921, Accuracy = 0.836) (**Table 3**). This was the best-performing model and outperformed the SR model. The sixteen radiomic features that formed the best radiomics signature included nine SR features, three GGR features, and four PR features (**Figure 4C**). **Supplementary Table S3** provides details on the performance of three machine learning classifiers using radiomic features from multiple ROIs.

### Model performance comparison

The GGR+SR+PR model did not demonstrate a significant difference in AUC value from the GGR+SR model (P = 0.646) or SR model (P = 0.752) in the external test group. The AUC value of the GGR+SR+PR model was significantly superior to that of all other models, as detailed in **Table 3**. The DCA presented in **Figure 4D** shows that the GGR+SR+PR model achieved greater net benefit within more threshold probabilities than did the clinical model.

### Model interpretability with SHAP

**Figure 5:**
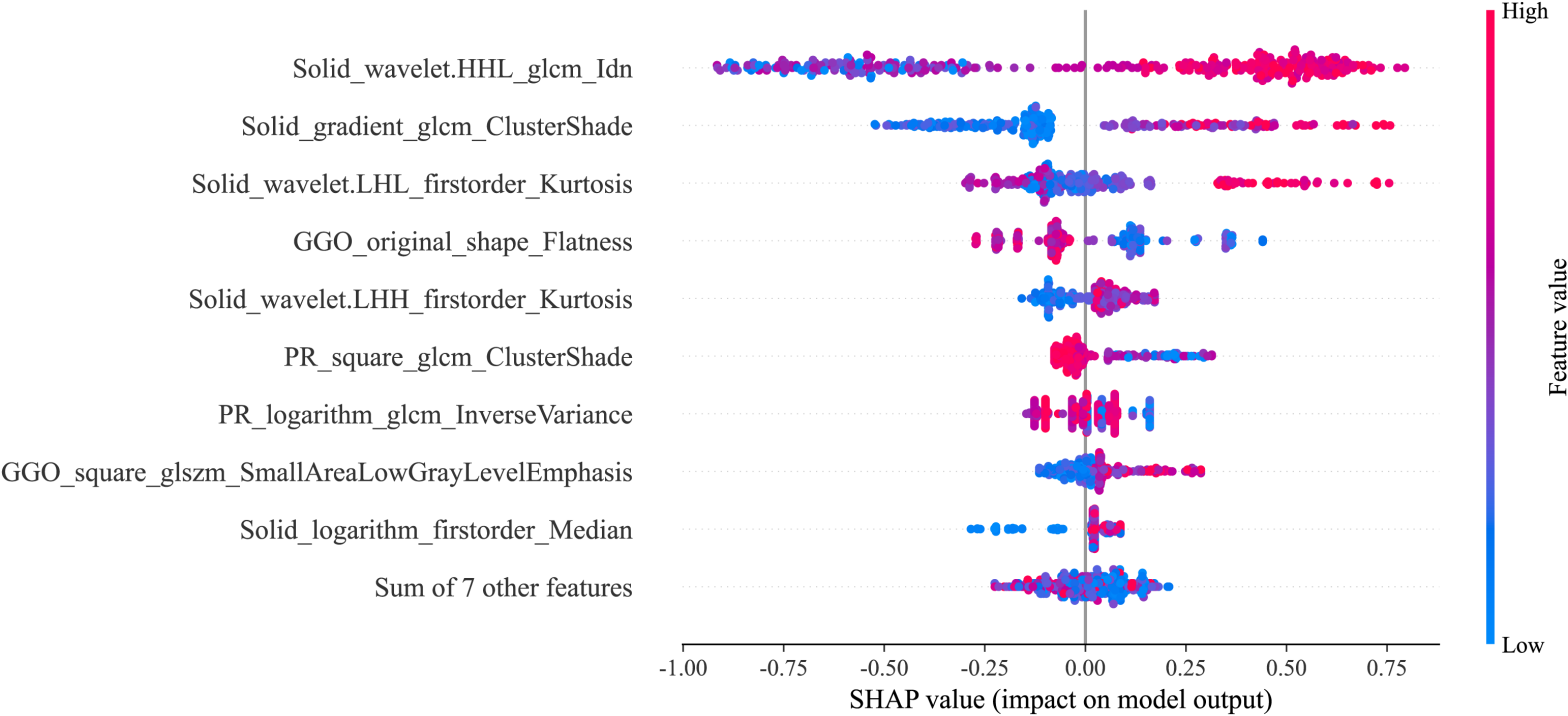
SHAP summary plot of best-performing model.

**Figure 6:**
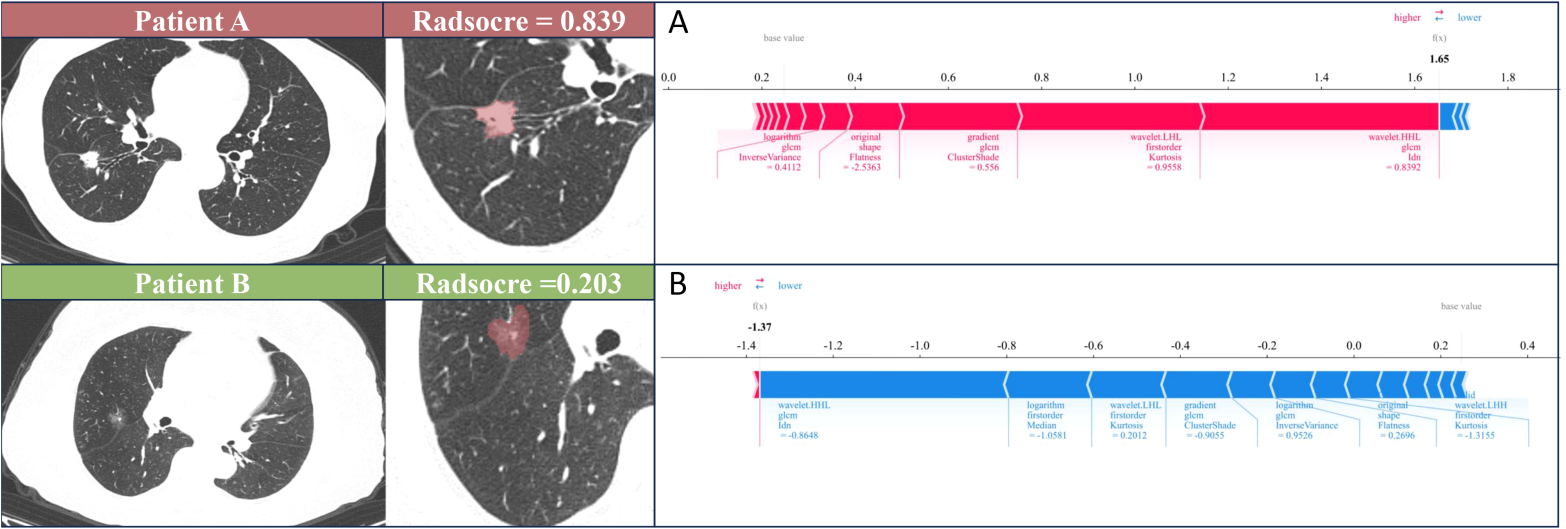
SHAP force plot illustrates the reasoning process behind two representative cases. **(A)** The STAS status was positive of patient A. **(B)** The STAS status was negative of patient B. The base value is the predicted value when no input is provided to the model, while the bold numbers represent the probability predicted value (f(x)). Blue features represent decreased risk, while red features represent increased risk. The length of the arrow reflects the degree of influence on the prediction. The longer the arrow, the greater the effect.

In **Figure 5**, it can be observed that wavelet-HHL_glcm_Idn from SR played a vital role in the best radiomics model’s differentiation of STAS status. The color map indicates a positive correlation between the SHAP value of this feature and the model’s output. For the individual sample predictions, we randomly chose two patients to make the force plot (**Figure 6**). Each forecast began with a base value of 0.247, representing the average SHAP value of all predictions. In **Figure 6A**, the SHAP value of the patient is 1.65, which exceeds the base value and results in a prediction of positive for STAS. Conversely, in **Figure 6B**, the patient’s SHAP value was −1.37, suggesting a prediction of STAS negative.

## Discussion

In this study, we constructed several radiomics signatures using separate regions of nodules (ground-glass, solid, gross, and perinodular) individually and in combination, and explored the potential of these radiomic features for predicting STAS status in lung adenocarcinoma. The GGR+SR+PR model exhibited the highest performance, achieving AUCs of 0.959 and 0.840, respectively, in the training and external test groups, indicating its potential as a valuable preoperative tool for clinical decision making.

STAS represents a crucial risk factor affecting patient survival and postoperative recurrence (6). The preoperative accurate prediction of the STAS status in lung adenocarcinoma is conducive to the selection of surgical approaches. Multiple studies have demonstrated that the solid tumor component is associated with STAS status (16, 33). In our study, the CTR emerged as an independent predictor of STAS, indicating that tumors positive for STAS tend to have a greater proportion of solid components, aligning with their observed findings. Additionally, the present research showed that CEA levels were frequently elevated in patients with STAS-positive lung adenocarcinoma, which is consistent with previous study finding (23). This suggests that this tumor marker may serve as an important indicator for STAS in lung adenocarcinoma. However, this factor was not an independent risk factor for STAS in our study.

Previous studies have used radiomics to assess STAS. Jiang et al. (21) built a CT-based radiomics signature using the random-forest classifier that predicted STAS with a specificity of 0.588, sensitivity of 0.880, and AUC of 0.754, demonstrating good diagnostic capability. However, this study was a single-center study. Chen et al. (34) extracted radiomic features from 233 stage I lung adenocarcinoma and constructed a CT-based predictive model for STAS that achieved AUCs of 0.63 and 0.69 in the internal and external validation cohorts, respectively. However, this model showed moderate predictive performance. Moreover, these studies only extracted radiomic features from the gross tumor region. In our study, we extracted radiomic features from different regions of nodules, and found that the radiomics model based on combined features from GGR, SR, and PR showed good discrimination ability (AUC = 0.840) and was the best-performance model. By analyzing these radiomic features, we observed that the predictive performance of the GGR+SR model (AUC = 0.832) outperformed the GNR model (AUC = 0.674) when applied to the external test cohort. This result may indicate that the combined features of GGR and SR had additional value compared with the GNR features alone. Additionally, we also found that the predictive performance of the SR model (AUC = 0.831) was superior to that of the GGR model (AUC = 0.659) and GNR model (AUC = 0.674). Radiomics can reflect the heterogeneity of tumors, this result demonstrates that radiomic features from SR were useful in predicting STAS, reflecting the relationship between STAS and the solid components of the tumor. STAS is mainly distributed around the primary tumor lesion, and studies (23, 35) have demonstrated that radiomic features extracted from the peritumoral area were feasible for predicting STAS status. In our study, the prediction performance of the model was improved when combined radiomic features from SR, GGR and PR, indicating PR features had a certain predictive value.

Effective and dependable machine learning classifiers aid in enhancing the successful use of radiomics within clinical practice (36). To enhance the robustness of our research, we chose 3 machine learning classifiers. The primary benefit of LightGBM is the substantial speed-up in the training process, which leads to the creation of more effective models (37, 38). Our best-performing radiomics signature contained a high proportion of SR features (9/16), and these had significant predictive weight, suggesting that the solid regions contain important information reflecting the STAS status. This further illustrates the association between STAS and solid components. The SHAP analysis provides explanations and visualizations for the LighGBM model through SHAP summary plots and SHAP force plots. In this study, we found that the wavelet-HHL_glcm_Idn feature based on SR was the top feature that contributed the most to the best radiomics signature. Wavelet features can reflect heterogeneity within the tumor and better represent the image information (39). A previous study showed that wavelet features are capable of effectively predicting the STAS status in lung adenocarcinoma (40).

Our research is subject to several limitations. First, this study was based on retrospective analysis, which inherently has selection bias. Second, employing a manual and semi-automatic method for segmenting the ROI introduces a level of subjectivity that could influence the findings. Third, the sample size is relatively small and is need a larger prospective study to validate our findings. Fourth, this study only analyzed the perinodular area extending 5 mm from the edge of the nodule to the periphery, and a wider range of perinodular area needs to be further explored in the future. Finally, this study utilized only non-enhanced CT images, and future work should integrate contrast-enhanced CT to further explore the value of radiomics for predicting STAS.

## Conclusion

In conclusion, CT radiomic features based on SR can contribute to identifying the STAS status in lung adenocarcinoma. Combined radiomic features from ground-glass, solid, and perinodular areas of PSNs enhances the prediction ability of the model.

## Data Availability

The original contributions presented in the study are included in the article/**Supplementary Material**. Further inquiries can be directed to the corresponding authors.

## Supplementary material

Supplementary Table 1 The parameters of CT scans.

Supplementary Table 2 Performance of three machine learning classifiers with reference to individual ROIs.

Supplementary Table 3 Performance of three machine learning classifiers with reference to multiple ROIs.
