# Supplementary material for: To predict the spread through air spaces in lung adenocarcinoma using radiomic features from different regions of part-solid nodules: a multicenter study
Source: Front Oncol. 2025 Oct 31;15:1700843. doi: 10.3389/fonc.2025.1700843 (PMC12615219; doi:10.3389/fonc.2025.1700843)
Supplement: Supplementary Table 1 — The parameters of CT scans. [file Table1.docx]

**Table S1** The parameters of CT scans

| CT scan device | Somatom Definition Flash dual-source, Siemens Healthcare | Aquilion ONE, TOSHIBA | Discovery 750, GE Healthcare | Somatom Sensation Cardiac 64, Siemens Healthcare |
| --- | --- | --- | --- | --- |
| Detector | 128×0.625mm | 64×0.625mm | 64×0.625mm | 64×0.625mm |
| Matrix | 512×512 | 512×512 | 512×512 | 512×512 |
| Reconstruction slice thickness | 1mm | 1mm | 1.25mm | 0.625mm |
| Pitch | 1.2 | 0.9 | 0.9 | 0.9 |
| Tube current | automatic tube current modulation | automatic tube current modulation | automatic tube current modulation | automatic tube current modulation |
| Tube voltage | 120kV | 120kV | 120kV | 120kV |

Note: kV = kilovolt; mA, milliampere.

**Table S2** Performance of three machine learning classifiers with reference to individual ROIs

| Region | Classifier | Training cohort | |  | External test cohort | |
| --- | --- | --- | --- | --- | --- | --- |
|  |  | AUC (95% CI) | Accuracy |  | AUC (95% CI) | Accuracy |
| GNR | LR | 0.765 (0.703-0.827) | 0.713 |  | 0.674 (0.561-0.787) | 0.691 |
|  | SVM | 0.781 (0.719-0.843) | 0.717 |  | 0.648 (0.531-0.766) | 0.636 |
|  | LightGBM | 0.809 (0.754-0.865) | 0.709 |  | 0.632 (0.516-0.747) | 0.627 |
| SR | LR | 0.812 (0.757-0.868) | 0.749 |  | 0.777 (0.671-0.882) | 0.800 |
|  | SVM | 0.823 (0.767-0.880) | 0.780 |  | 0.774 (0.666-0.882) | 0.755 |
|  | LightGBM | 0.926 (0.894-0.958) | 0.834 |  | 0.831 (0.741-0.920) | 0.755 |
| GGR | LR | 0.743 (0.677-0.809) | 0.709 |  | 0.617 (0.497-0.738) | 0.700 |
|  | SVM | 0.738 (0.671-0.805) | 0.704 |  | 0.659 (0.540-0.779) | 0.691 |
|  | LightGBM | 0.922 (0.887-0.958) | 0.848 |  | 0.509 (0.391-0.627) | 0.491 |
| PR | LR | 0.782 (0.722-0.843) | 0.735 |  | 0.628 (0.514-0.742) | 0.500 |
|  | SVM | 0.870 (0.821-0.918) | 0.812 |  | 0.644 (0.531-0.756) | 0.518 |
|  | LightGBM | 0.917 (0.880-0.953) | 0.865 |  | 0.648 (0.523-0.773) | 0.682 |

Note: AUC, area under the receiver operating characteristic curve; CI, confidence interval; GNR, gross nodule region; SR, solid region; GGR, ground-glass opacity region; PR, perinodular region; LR, logistic regression; SVM, support vector machine; LightGBM, light gradient boosting machine.

**Table S3** Performance of three machine learning classifiers with reference to multiple ROIs

| Region | Classifier | Training cohort | |  | External test cohort | |
| --- | --- | --- | --- | --- | --- | --- |
|  |  | AUC (95% CI) | Accuracy |  | AUC (95% CI) | Accuracy |
| GGR+SR | LR | 0.823 (0.769-0.876) | 0.753 |  | 0.768(0.666-0.871) | 0.700 |
|  | SVM | 0.862 (0.814-0.910) | 0.789 |  | 0.755 (0.646-0.863) | 0.745 |
|  | LightGBM | 0.936 (0.907-0.966) | 0.857 |  | 0.832 (0.743-0.920) | 0.836 |
| GNR+PR | LR | 0.801 (0.744-0.859) | 0.749 |  | 0.714 (0.603-0.825) | 0.600 |
|  | SVM | 0.830 (0.775-0.885) | 0.771 |  | 0.655 (0.544-0.767) | 0.664 |
|  | LightGBM | 0.867 (0.821-0.913) | 0.776 |  | 0.692 (0.574-0.811) | 0.691 |
| GGR+SR+PR | LR | 0.865 (0.818-0.912) | 0.785 |  | 0.747 (0.632-0.862) | 0.718 |
|  | SVM | 0.896 (0.851-0.940) | 0.843 |  | 0.731 (0.619-0.843) | 0.709 |
|  | LightGBM | 0.959 (0.936-0.982) | 0.901 |  | 0.840 (0.758-0.921) | 0.836 |

Note: AUC, area under the receiver operating characteristic curve; CI, confidence interval; GGR+SR, ground-glass opacity region and solid region; GNR+PR, gross nodule region and perinodular region; GGR+SR+PR, ground-glass opacity region, solid region, and perinodular region; LR, logistic regression; SVM, support vector machine; LightGBM, light gradient boosting machine.
